# Effect of Cu/Mn-Fortification on In Vitro Activities of the Peptic Hydrolysate of Bovine Lactoferrin against Human Gastric Cancer BGC-823 Cells

**DOI:** 10.3390/molecules24071195

**Published:** 2019-03-27

**Authors:** Li-Ying Bo, Tie-Jing Li, Xin-Huai Zhao

**Affiliations:** 1 Key Laboratory of Dairy Science, Ministry of Education, Northeast Agricultural University, Harbin 150030, China; Boliying1746@126.com; 2 Department of Food Science, Northeast Agricultural University, Harbin 150030, China

**Keywords:** lactoferrin hydrolysate, copper, manganese, gastric cancer cells, anti-cancer activity, molecular mechanism

## Abstract

Bovine lactoferrin hydrolysate (BLH) was prepared with pepsin, fortified with Cu^2+^ (Mn^2+^) 0.64 and 1.28 (0.28 and 0.56) mg/g protein, and then assessed for their activity against human gastric cancer BGC-823 cells. BLH and the four fortified BLH products dose- and time-dependently had growth inhibition on the cells in both short- and long-time experiments. These samples at dose level of 25 mg/mL could stop cell-cycle progression at the G0/G1-phase, damage mitochondrial membrane, and induce cell apoptosis. In total, the fortified BLH products had higher activities in the cells than BLH alone. Moreover, higher Cu/Mn fortification level brought higher effects, and Mn was more effective than Cu to increase these effects. In the treated cells, the apoptosis-related proteins such as Bad, Bax, p53, cytochrome c, caspase-3, and caspase-9 were up-regulated, while Bcl-2 was down-regulated. Caspase-3 activation was also evidenced using a caspase-3 inhibitor, z-VAD-fmk. Thus, Cu- and especially Mn-fortification of BLH brought health benefits such as increased anti-cancer activity in the BGC-823 cells via activating the apoptosis-related proteins to induce cell apoptosis.

## 1. Introduction

Dietary proteins provide both essential amino acids and energy for the body, and also have several health benefits by the release of so-called bioactive peptides [1], because these peptides have various physiological functions such as anti-cancer, anti-hypertensive, anti-oxidant, mineral-binding, and other effects [2,3]. The solid fraction from yogurt exerts growth inhibition on initial tumor cells, while the peptide fraction from algae protein has anti-cancer activity against the gastric cancer AGS cells through arresting the cells in the post-G1-phase [4,5]. An important Fe-binding protein lactoferrin (LF) and its derivatives have also been assessed for their bio-activities. LF and a LF derivative lactoferricin B have anti-cancer activities in the gastric cancer SGC-7901, AGS cells and oral squamous cell carcinoma [6,7,8]. Lactoferricin B is also well-known for its anti-bacterial effect against a wide variety of Gram-positive and Gram-negative bacteria [9,10]. From a chemical point of view, proteins have various functional groups such as −OH, −SH, −NH, etc., and thus can interact with some macro-elements and trace elements, resulting in changed nutritive values and bio-activities. For LF, Cu supplementation increases immuno-modulation in both murine splenocytes and RAW264.7 macrophages, while Fe addition can enhance growth inhibition and apoptosis induction in the HepG2 cells infected with HBV [11,12]. LF in the stomach is digested by a proteolytic enzyme pepsin; after that, the yielded LF hydrolysate might also have opportunity to interact with other dietary components including those multivalent trace metal ions. To the best of our knowledge, very few data are available on the effect of interaction between LF hydrolysate and trace metals on anti-cancer activity of LF hydrolysate in some cancer cells, and such study clearly deserves consideration in the scientific community.

Both Cu and Mn are commonly regarded as essential elements to the body. Cu plays crucial roles in the functions of proteins and many enzymes involved in energy metabolism, DNA synthesis, and respiration [13]. For example, Cu is a critical cofactor of the well-known superoxide dismutase and cytochrome oxidase [14]. Mn is also necessary for a series of physiological processes such as the metabolism of carbohydrates, lipids, and amino acids, and has important role as the cofactor of several enzymes in metabolism in the brain [15]. Both Cu^2+^ and Mn^2+^ can complex with some organic materials, which have been studied for their anti-cancer, immune, and anti-oxidant effects [16,17]. Two Cu complexes can inhibi tumor cell growth, while the Mn complex of N-substituted di(picolyl)amine can inhibit the growth of both U251 and HeLa cells via interfering with mitochondrial functions [18,19]. When LF hydrolysate in the stomach interacts with Cu^2+^ or Mn^2+^, potential changes in its activity against gastric cancer BGC-823 cells are promising. However, to the best of our knowledge, these changes are still not assessed.

In our previous study [20], we used the well-differentiated gastric cancer AGS cells as model cells to evaluate the effects of Cu^2+^ and Mn^2+^ fortification on anti-cancer activity of a peptic bovine lactoferrin hydrolysate (namely BLH). Both Cu- and Mn-fortification were evident to increase BLH’s anti-cancer activity against the AGS cells, through two events: enhanced apoptosis induction and autophagy inhibition. However, it is regarded that higher degree of cancer cell differentiation generally accompanies lower degree of malignancy. The low-differentiated gastric cancer cells thus deserve another investigation. In the present study, the low-differentiated gastric cancer BGC-823 cells were used as model cells. BLH was also fortified with CuCl_2_ and MnSO_4_ of two levels, and then they were assessed and compared for their anti-cancer activity changes using growth inhibition, cell-cycle arrest, mitochondrial membrane disruption, and apoptosis induction as evaluation indices. Furthermore, expression changes of several apoptosis-related proteins were assayed to disclose possible molecular mechanism responsible for the anti-cancer activity changes of the Cu/Mn-fortified BLH.

## 2. Results

### 2.1. Chemical Features of LF, BLH, and Mixtures I–IV

In this study, the used bovine LF and BLH had protein contents of about 957.3 and 923.4 g/kg, and Fe contents of about 140.6 and 130.3 mg/kg (Table 1), respectively. Compared with bovine LF, BLH had higher −NH_2_ content (0.93 versus 0.49 mmol/g protein), due to the conducted peptic digestion. BLH was also measured with a DH value of 5.1 ± 0.1%. Due to Cu/Mn fortification, the prepared BLF-Cu mixtures (i.e., Mixtures I−II) or BLF-Mn mixtures (i.e., Mixtures III–IV) in this study contained more Cu or Mn than BLH. Thus, activity changes of these mixtures in the assessed BGC-823 cells mainly arose from the fortified Cu or Mn ions.

### 2.2. Growth Inhibition of BLH and Mixtures I–IV on the BGC-823 Cells

**Figure 1:**
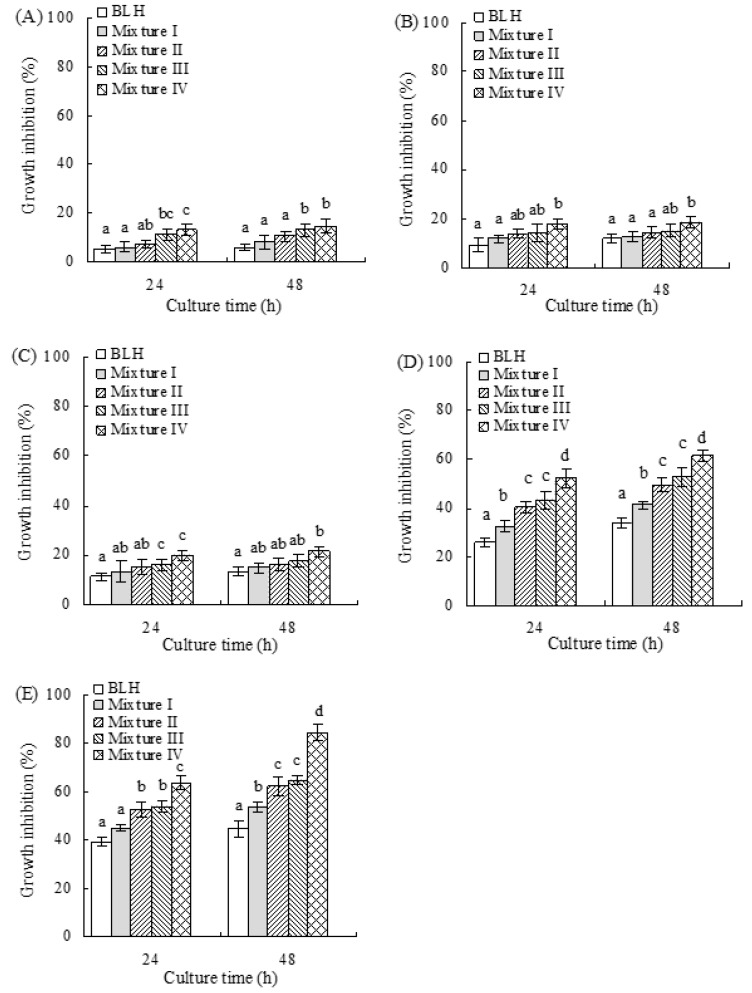
Growth inhibition of BLH and Mixtures I–IV at five dose levels on the BGC-823 cells with treatment times of 24 and 48 h. Mixtures I−II represent bovine lactoferrin hydrolysate (BLH) fortified with Cu^2+^ at 0.64 and 1.28 mg/g protein, while Mixtures III–IV represent BLH fortified with Mn^2+^ at 0.28 and 0.56 mg/g protein, respectively. (**A**–**E**) The mixtures were used at concentrations of 10, 15, 20, 25, and 30 mg/mL, respectively. Different letters like a, b, c, and d above the columns in the same culture time show that the means of different groups were significantly different (*p* < 0.05) by one-way analysis of variance.

In this study, 5-FU as positive control could obviously inhibit the growth of BGC-823 cells: at 200 μmol/L, it resulted in growth inhibition values of 43.5 (24 h) and 58.7% (48 h) (Figure 1). BLH and its mixtures also exerted growth inhibition on the cells (Figure 1). BLH time- and dose-dependently showed growth inhibition values of 5.3–44.7%. Mixtures I–IV also time- and dose-dependently inhibited cell growth, and were more effective than BLH, bringing increased growth inhibition values ranging from 6.3% to 84.5%. Mixtures III–IV showed higher inhibition on the cells than Mixtures I−II (growth inhibition values 11.3–84.5% versus 6.3–62.3%). It was also seen that Mixture I (or Mixture III) had weaker growth inhibition than Mixture II (or Mixture IV), based on these measured growth inhibition values. These results indicated that it was the fortified Cu and especially Mn conferred BLH with higher growth inhibition on the cells, while higher Cu/Mn fortification levels led to higher inhibitory effect. All assessed samples at dose levels other than 25 mg/mL gave too weak or too strong growth inhibition on the cells; thus, they were only used at 25 mg/mL with treatment time of 24 h in later assays.

**Figure 2:**
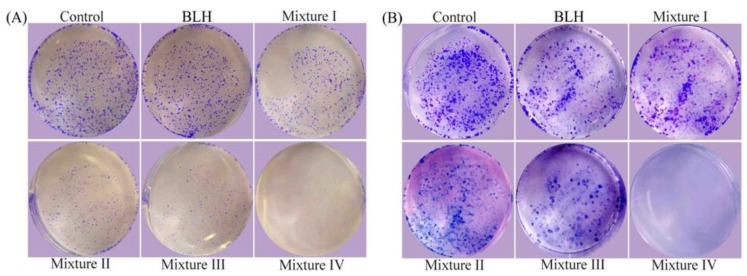
Long-term anti-proliferation of BLH and Mixtures I–IV on the BGC-823 cells with culture times of: 10 days (**A**); and 20 days (**B**).

When BLH and Mixtures I–IV were used at dose level of 25 mg/mL to assay their long-term growth inhibition on the cells (10 and 20 days), the results showed that Mixtures I–IV also had higher anti-proliferative effects on the cells than BLH (Figure 2). Based on the observed sizes and numbers of cell colonies, it was evident that Mixtures III–IV possessed higher activity than Mixtures I−II, while Mixture IV (or Mixture II) had higher effect than Mixture III (or Mixture I). That is, Mn was more effective than Cu to enhance long-term growth inhibition of BLH, and higher Cu/Mn fortification levels also resulted in higher long-term anti-proliferation.

### 2.3. Effects of BLH and Mixtures I–IV on Cell-Cycle Progression of the BGC-823 Cells

**Figure 3:**
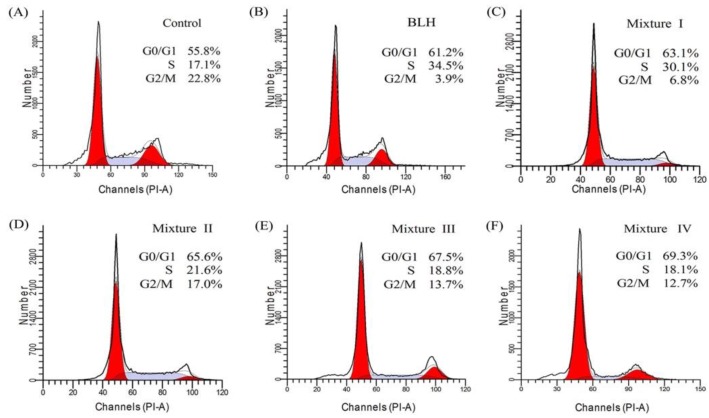
Cell-cycle distribution of the BGC-823 cells: without any treatment (**A**); or treated with BLH (**B**) and Mixtures I–IV (**C**–**F**) at dose level of 25 mg/mL.

To further investigate whether BLH and Mixtures I–IV might cause cell growth inhibition via disturbing cell-cycle progression, flow cytometry analysis was done to detect cell-cycle distribution. Mixtures I–IV with treatment time of 24 h resulted in higher cell proportions at the G0/G1-phase than BLH did (63.1−69.3% versus 61.2%) (Figure 3). Of note, the cells treated by Mixtures I−II or Mixtures III–IV had different G0/G1-phase proportions (63.1−65.6% versus 67.5−69.3%). Mixtures I–IV were thus more efficient than BLH to arrest cell-cycle progression at the G0/G1-phase. Overall, Mn fortification led to greater cell-cycle arrest than Cu fortification, and higher Cu/Mn fortification level caused greater cell-cycle arrest at the G0/G1-phase. It is thus concluded that Cu and especially Mn endowed BLH with higher ability to stop cell-cycle progression at the G0/G1-phase, and thereby caused cell growth inhibition.

### 2.4. Apoptosis Induction of BLH and Mixtures I–IV to the BGC-823 Cells

**Figure 4:**
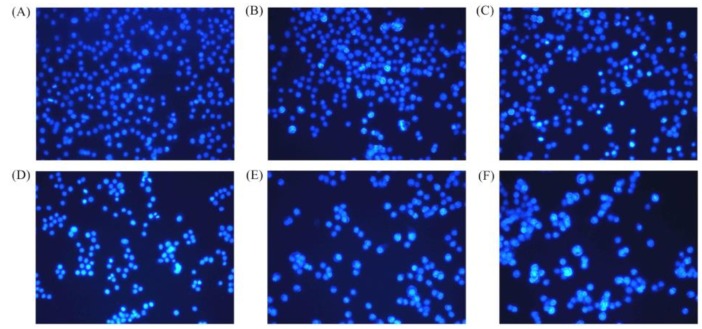
Observed morphology of the BGC-823 cells: without any treatment (**A**); or treated with BLH (**B**) and Mixtures I–IV (**C**–**F**) at dose level of 25 mg/mL by a fluorescence microscope at 200× magnification.

The classic Hoechst 33258 staining was used to observe the morphologic features of the BGC-823 cells exposed to BLH and Mixtures I–IV with treatment time of 24 h (Figure 4), to further disclose briefly if these samples had potential apoptosis induction to the cells. The control cells without any sample treatment had many cells in the observation vision; moreover, most of the control cells were observed to be dimly blue but only a few cells were apoptotic cells (Figure 4A). The cells exposed to BLH and especially Mixtures I–IV had decreased cell numbers in the observation vision, and increased numbers of apoptotic cells (brilliant blue together with chromatin condensation and nuclear fragmentation) were also observed (Figure 4B–F). These results suggest that BLH and Mixtures I–IV could cause cell apoptosis.

**Figure 5:**
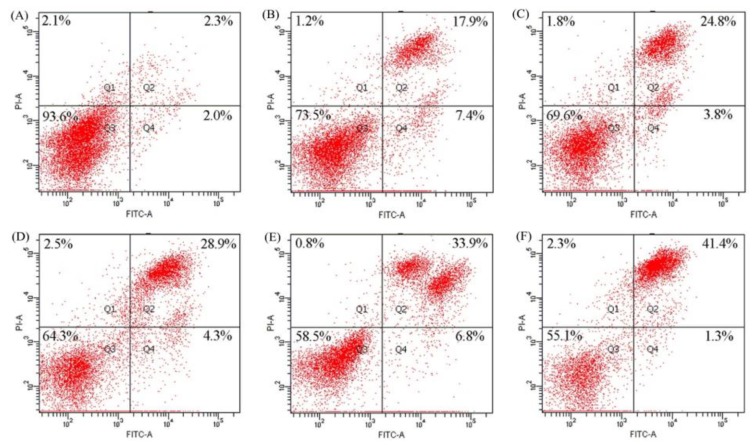
Cell proportions of the BGC-823 cells: without any treatment (**A**); or treated with BLH (**B**) and Mixtures I–IV (**C**–**F**) at dose level of 25 mg/mL. Q1−Q4 represent necrotic, late apoptotic, intact, and early apoptotic cells, respectively.

Apoptosis induction of BLH and Mixtures I–IV in the BGC-823 cells was then assayed by the classic flow cytometry technique, based on measured total apoptotic cell proportions (i.e., Q2 + Q4). The results (Figure 5) show that these samples all had apoptosis induction in the treated cells. The control cells had total apoptotic proportion of 4.3%. The cells exposed to Mixtures I–IV showed higher total apoptotic proportions (28.6%, 33.2%, 40.7%, and 42.7%, respectively) than those exposed to BLH alone (25.3%). Mixture IV (or Mixture II) more obviously caused cell apoptosis than Mixture III (or Mixture I). It was thus proposed that Mn fortification was more effective than Cu fortification to endow BLH with higher apoptosis induction, and higher Cu/Mn fortification level also brought higher activity. For these assessed samples, the order of apoptosis induction was completely consistent with the order of cell-cycle arrest (Figure 5), suggesting that both apoptosis induction and cell-cycle arrest contributed to the assayed growth inhibition.

### 2.5. Mitochondrial Membrane Disruption of the BGC-823 Cells by BLH and Mixtures I–IV

**Figure 6:**
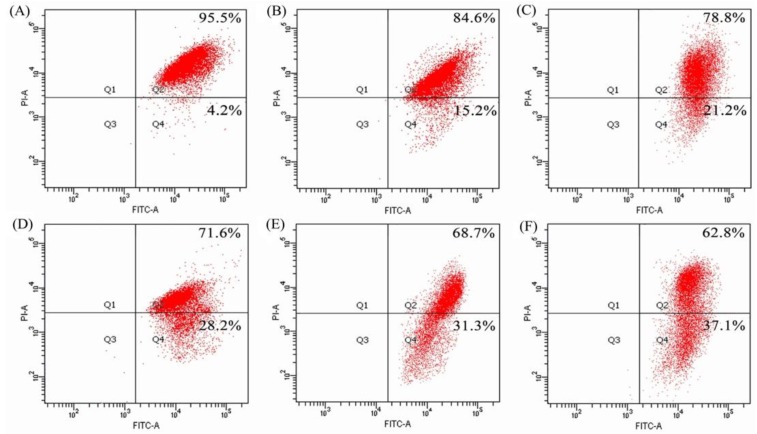
MMP loss of the BGC-823 cells: without any treatment (**A**); or treated with BLH (**B**) and Mixtures I–IV (**C**–**F**) at dose level of 25 mg/mL.

Mitochondrial membrane potential (MMP) of the BGC-823 cells exposed to BLH and Mixtures I–IV were analyzed using flow cytometry and JC-1 dye staining, to further verify whether the treated cells had mitochondrial dysfunction. The cells treated by BLH had decreased MMP (cell proportion of red fluorescence 84.6%, Figure 6B), compared with the control cells without sample treatment (95.5%, Figure 6A). Moreover, the cells treated with Mixtures III–IV had lower cell proportions of red fluorescence (68.7% and 62.8%, Figure 6E,F) than those treated with Mixtures I−II (red fluorescence of 78.8% and 71.6%, Figure 6C,D). Mixtures I–II and especially Mixtures III–IV thereby brought greater MMP loss in the treated cells. It was thus demonstrated that these samples caused mitochondrial membrane disruption, and then led to the release of cytochrome c to trigger cell apoptosis. It was also seen from these measured data that Mn fortification was more efficient than Cu fortification to induce MMP loss, and higher Cu/Mn fortification levels brought increased MMP loss.

### 2.6. Expression Changes of Apoptosis-related Proteins in the BGC-823 Cells

**Figure 7:**
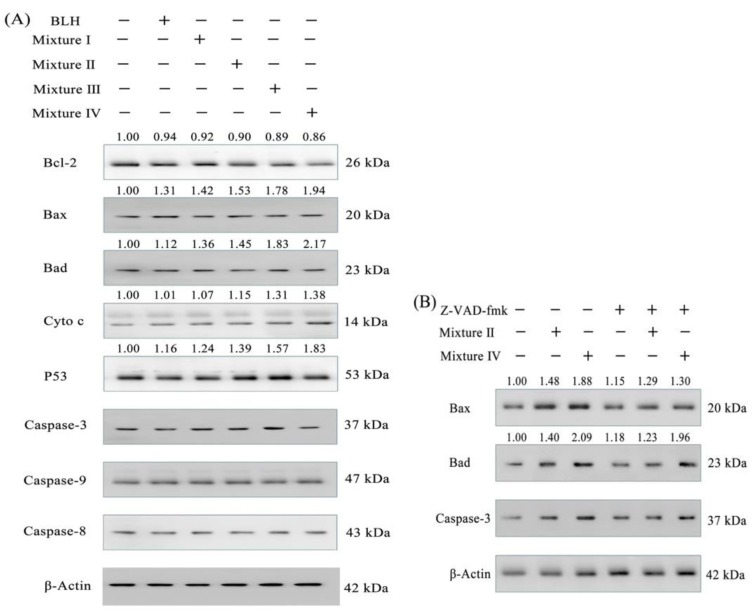
Expression changes of the apoptosis-related proteins in the BGC-823 cells treated with BLH and the Mixtures I–IV (**A**), respectively or treated with Mixture II or Mixture IV in the absence or presence of a caspase-3 inhibitor z-VAD-fmk (**B**).

**Figure 8:**
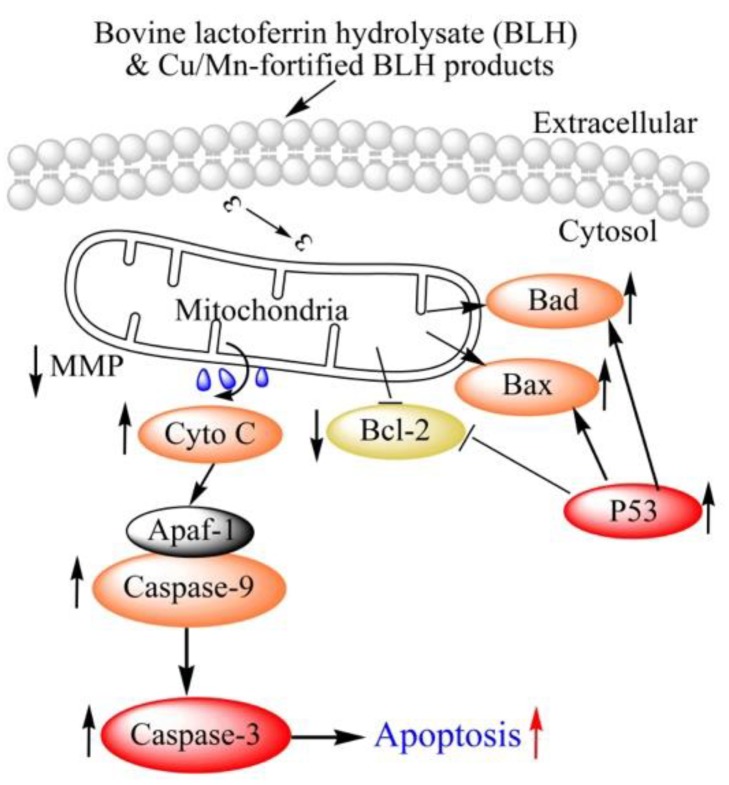
Proposed mechanism responsible for apoptosis induction of BLH and its Cu/Mn mixtures.

Serial Western-blot assays were done to evaluate expression levels of seven proteins in the treated cells that have been classified as apoptosis-related proteins. In total, BLH and Mixtures I–IV in the cells could up-regulate Bax, Bad, p53, and cytochrome c expression and down-regulate Bcl-2 expression, together with caspase-3 and caspase-9 activation; however, these samples did not cause clear change in caspase-8 expression (Figure 7A). Mn fortification was more efficient than Cu fortification to regulate the expression of these proteins. Mixtures I–IV thus had enhanced anti-cancer activities against the BGC-823 cells than BLH alone, mainly via mediating the expression of these apoptosis-related proteins. Using the caspase-3 inhibitor z-VAD-fmk in the cells could provide further evidence (Figure 7B). When the cells were treated by the z-VAD-fmk, Mixture II and especially Mixture IV showed the ability to increase the expression of Bad (relative expression folds 1.29 and 1.30 vs. 1.15) and Bax (relative expression folds 1.23 and 1.96 vs. 1.18). These results suggest that both Mixture II and Mixture IV indeed were able to induce caspase-3 activation or cell apoptosis. BLH and Mixtures I–IV were thus suggested to induce cell apoptosis via the caspase-3-dependent pathway (Figure 8).

## 3. Discussion

Food hydrolysates possess in vitro anti-cancer activities to many cancer cells such as PC-3, DU-145, H-1299, and Hela cells [21,22,23]. Bovine LF as one of the most important bioactive proteins in milk has anti-cancer activity to cancer cells, but is regarded to be harmless to normal cells [24,25,26]. It has been demonstrated that bovine BLH has growth inhibition in gastric cancer and oral squamous cell carcinoma [7,8], can inhibit metastasis of liver and lung cancer cells in the mice [27], and displays anti-cancer effects in colon cancer cells [28]. In this study, BLH and the Cu/Mn-fortified Mixtures I–IV all had anti-cancer activities against the BGC-823 cells with clear growth inhibition, cell-cycle block, and apoptosis induction. The present results are thus consistent with the reported ones. When BLH was fortified with Cu or Mn ions, the resultant mixtures had enhanced anti-cancer effects in the cells. Similarly, the Fe-fortified bovine LF has enhanced growth inhibition on the HepG2 cells infected with HBV [12]. Two previous studies also verify that catechin, epicatechin, epigallocatechin, and particularly epigallacatechin-3-gallate in the presence of Cu can induce apoptosis of a breast cancer cell line MDA-MB-231 [29,30]. It is reasonable that the fortified Cu/Mn contributed these enhanced effects. Mn was always more efficient than Cu to increase these measured effects, which is important but was unsolved in the present study.

In general, protein hydrolysates exert anti-cancer effects via different pathways including anti-proliferation, cell-cycle arrest, apoptosis induction, and others. Rapid growth of cancer cells is achieved by cell continuous division, while cell-cycle is a programmed process of cell division. Thus, stopping cell-cycle progression at a certain cell phase is an important way to inhibit the growth of cancer cells [31]. The hydrolysates derived from donkey milk thus can arrest cell-cycle progression of human lung cancer A549 cells at the G0/G1-phase, while those from roe also can arrest cell-cycle of human oral cancer cells Ca9-22 and CAL27 at the sub-G1-phase [32,33]. Meanwhile, cell apoptosis is a critical mechanism of programmed cell death and, therefore, the induced cell apoptosis is a promising strategy for cancer treatment [34]. Protein hydrolysates derived from giant grouper (*Epinephelus Lanceolatus*) can induce apoptosis of human oral cancer cells, while those from tuna cooking juice induce apoptosis in human breast cancer MCF-7 cells [33,35]. These mentioned findings all support that BLH and Mixtures I–IV had cell-cycle arrest and apoptosis induction, and thereby led to growth inhibition in the cells.

In this study, the treated cells had changed morphologic features and especially MMP loss. This fact suggests potential disruption of mitochondrial membrane and subsequently release of cytochrome c. BLH and Mixtures I–IV thus could induce the apoptosis of the BGC-823 cells via the classic caspase-3-dependent pathway (or mitochondrial pathway). Cytochrome c released (a positive event of cell apoptosis) from the mitochondria into the cytosol activates Apaf-1 and caspase-9, leading to caspase-3 activation and thereby cell apoptosis [36]. Apoptosis of cancer cells requires effective activation of a tumor suppressor p53 [37]. P53 is able to up-regulate pro-apoptotic proteins Bax and Bad, resulting in the increased permeability of mitochondrial membrane, cytochrome c release, and the activation of apoptogenic factors apaf-1. However, another anti-apoptotic protein Bcl-2 has a function to reduce cytochrome c release, which can be suppressed by p53 [38]. The peptides from rapeseed can up-regulate p53 and Bax but down-regulate Bcl-2 expression in HepG2 cells, while rice protein hydrolysates can induce H9c2 myocardiocytes apoptosis through the Bcl-2/Bax pathway [39,40]. More importantly, a previous study demonstrating a short-term cooperation of 3,4-dihydroxy-trans-stilbene and exogenous Cu also showed preferential apoptosis induction of HepG2 cells via mitochondria apoptosis pathway [41]. In this study, these assessed samples up-regulated the pro-apoptotic proteins Bad, Bax, and p53 but down-regulated the anti-apoptotic protein Bcl-2, and then increased cytochrome c release in the cytosol, which subsequently triggered the activation of caspase-9 and caspase-3 as well as cell apoptosis. However, caspase-8 expression, which represents the activation of the extrinsic apoptosis pathway, had no significant change in the cells (Figure 6 and Figure 7). This fact demonstrated that BLH and its fortified mixtures only activated the intrinsic but not extrinsic apoptosis pathway in the BGC-823 cells. Z-VAD-fmk as a classic caspase-3 inhibitor can suppress caspase-3 activation and inhibit the thapsigargin-induced cell death in human breast cancer cells MDA-MB-468 [42]. In this study, both Mixture II and Mixture IV decreased the suppression of z-VAD-fmk on caspase-3 activation via enhancing Bad and Bax expression (Figure 6), verifying that the disclosed apoptosis mechanism indeed was a caspase-3-dependent pathway. Mixtures I−II and especially Mixtures III–IV led to greater expression regulation on these apoptosis-related proteins than BLH did, and therefore exerted higher anti-cancer activity in the cells. However, whether BLH and the fortified mixtures could display anti-cancer effects via other pathways or mechanisms should be disclosed in the future. Moreover, whether these samples might have anti-cancer effects on other cancer cells is still unsolved.

## 4. Materials and Methods

### 4.1. Materials

Bovine LF was purchased from MILEI Gmbh (Leutkirch, Germany). The Dulbecco’s modified Eagle’s medium (DMEM) and porcine gastric mucosa pepsin (CAS: 9001-75-6) were purchased from Sigma-Aldrich Co. Ltd. (St. Louis, MO, USA), while the fetal bovine serum (FBS) was bought from Wisent Inc. (Montreal, QC Canada). Dextran T-70, phosphate-buffered saline (PBS), and Hoechst 33258 dye were bought from Solarbio Science and Technology Co. Ltd. (Beijing, China). 5-Fluorouracil (5-FU) was bought from Jinyao Pharmaceutical Co. Ltd. (Tianjin, China). Annexin V-FITC Apoptosis Detection Kit, Cell Cycle Analysis Kit, BCA Protein Assay Kit, RIPA Lysis Buffer, Hoechst 33258 dye, crystal violet dye, JC-1 dye, and phenylmethanesulfonyl fluoride (PMSF) were all purchased from Beyotime Institute of Biotechnology (Shanghai, China). Cell Counting Kit-8 (CCK-8) was bought from Dojindo Molecular Technologies, Inc. (Kyushu, Japan). Caspase-3 inhibitor z-VAD-fmk, primary anti-bodies (β-actin, caspase-3, caspase-9, caspase-8, Bad, Bax, p53, cytochrome c, and Bcl-2), and secondary anti-body were bought from Cell Signaling Technology, Inc. (Boston, MA, USA). Other chemicals used in this study were analytical grade. Ultrapure water was generated from Milli-Q Plus (Millipore Corporation, New York, NY, USA), and used in this study.

The BGC-823 cells were purchased from Cell Bank of the Chinese Academy of Sciences (Shanghai, China), and cultured at 37 °C in the DMEM with 10% FBS, 100 units/mL penicillin, and 100 μg/mL streptomycin, using a humidified incubator with 5% CO_2_.

### 4.2. Sample Preparation

BLH was prepared as previously described [43]. In brief, 5.0 g bovine LF was dissolved in 100 mL water, adjusted to pH 2.5 using 1 mol/L HCl, added with pepsin of 750 units/g protein, kept at 37 °C for 4 h, heated at 80 °C for 15 min to inactive pepsin, cooled to 20 °C, neutralized to 7.0 using 1 mol/L NaHCO_3_, and centrifuged at 12,000× *g* for 30 min at 4 °C. The collected supernatant (i.e., BLH) was freeze-dried with a freeze-dryer (ALPHA 1-4 LSCplus, Marin Christ, Osterode, Germany), ground into powder, and then stored at −20 °C until use.

BLH was dissolved in water, and added with CuCl_2_ (or MnSO_4_) solution to achieve final Cu (or Mn) levels of 0.64 and 1.28 (or 0.28 and 0.56) mg/g protein. Mixture I and Mixture II were designated as the Cu-fortified BLH with 0.64 and 1.28 Cu mg/g protein, while Mixture III and Mixture IV were designated as the Mn-fortified BLH with 0.28 and 0.56 Mn mg/g protein, respectively.

### 4.3. Sample Analyses

The protein contents of the samples were assayed using the Kjidahl method and a conversion factor of 6.38, while Fe content was detected using the *o*-phenanthroline method [44]. The content of free amino groups (−NH_2_) was measured using the *o*-pthaldialdehyde method together with standard L-leucine solutions of 0–36 mg/mL [45]. Degree of hydrolysis of BLH was calculated as previously described [46]. A spectrophotometer (UV-2401PC, Shimadzu, Kyoto, Japan) was used in these spectrometric analyses.

### 4.4. Assay of Cytotoxic Effect

The cells (2 × 10^4^ cells per well) were seeded in 96-well plates in 100 μL medium, and incubated for 24 h. The medium was replaced by 200 μL fresh medium containing BLH or Mixtures I–IV at dose levels of 10−30 mg/mL, followed by an incubation of 24 and 48 h and medium removal. CCK-8 solution of 100 μL (10 μL CCK-8 in 90 μL medium) was added into each well, followed by another incubation of 1.5 h. Optical density values were measured at 450 nm with a microplate reader (Bio-Rad Laboratories, Hercules, CA, USA), and used to calculate growth inhibition as previously described [20]. The cells exposed to 200 μmol/L 5-FU were designed as positive control, while those exposed to the media with 5% FBS were designed as negative control without any growth inhibition (i.e., 100% viability).

### 4.5. Colony Formation Assay

To evaluate long-term growth inhibition of these samples, the cells (1 × 10^3^ cells per well) were seeded in 6-well plates, and treated with the medium containing the assessed samples at dose level of 25 mg/mL for 24 h. Then, the medium with 5% FBS was replaced every 3 days. After an incubation of 10 or 20 days, the cells were fixed with methanol, stained with crystal violet dye, dried overnight, and then photographed with an EOS 6D Canon digital camera (Canon Inc., Tokyo, Japan).

### 4.6. Assay of Cell-Cycle Progression

The cells (1 × 10^6^ cells per dish) were seeded on 100-mm cell culture dish, incubated for 24 h with 10 mL medium, treated with 10 mL per dish fresh medium containing the assessed samples at dose level of 25 mg/mL for 24 h, harvested, washed twice with the cold PBS (10 mmol/L, pH 7.3), fixed with 70% cold ethanol by shaking once every 15 min overnight at 4 °C, washed with the cold PBS again, resuspended with binding buffer (500 μL), and stained with 10 μL RNase A and 25 μL propidium iodide (PI) for 30 min at 37 °C in the dark. The cells treated with the medium were designated as negative control. Cell proportions in the G0/G1-, S-, and G2/M-phases were measured using a flow cytometer (FACS Calibur, Becton Dickson, San Jose, CA, USA), and analyzed with the ModFit software (Verity Software House, Topsham, ME, USA).

### 4.7. Hoechst 33258 Staining

The cells (1 × 10^6^ cells per well) were seeded in 6-well plates with 2 mL medium, incubated for 24 h, and treated with medium containing the assessed samples at dose level of 25 mg/mL for 24 h. After removal of the medium, the cells were fixed by methanol for 5 min, washed twice with PBS, stained with Hoechst 33258 dye for 5 min in the dark at 22 °C, and observed under a fluorescence microscope (Type Eclipice-Ti-S, Nikon, Japan) with a magnification of 200×.

### 4.8. Assay of Mitochondrial Membrane Potential

Changes of mitochondrial membrane potential (MMP) of the treated cells were detected using the flow cytometer and JC-1 dye. The cells (5 × 10^5^ cells per well) were seeded in 6-well plates with 2 mL medium, cultured for 24 h, treated with the medium containing the samples at dose level of 25 mg/mL for 24 h, harvested, stained with JC-1 dye at 37 °C for 20 min, and then measured with the flow cytometer (FACS Calibur, Becton Dickson).

### 4.9. Assay of Apoptosis Induction

The cells (2 × 10^4^ cells per well) were seeded in 6-well plates with 2 mL medium, and incubated for 24 h. After medium removal, the cells were treated with the medium containing the samples at dose level of 25 mg/mL for 24 h. The cells treated with the medium consisting of 5% FBS served as negative control. After that, an AnnexinV-FITC/PI Apoptosis Detection Kit was used according to kit instruction. The cells were harvested, resuspended in 500 μL of the Annexin V-FITC binding buffer consisting of 5 μL Annexin V-FITC and 10 μL PI at 20 °C for 30 min in the dark, and assayed by the flow cytometry (FACS Calibur, Becton Dickson) to detect the intact (Q3), early apoptotic (Q4), late apoptotic (Q2), and necrotic (Q1) cell proportions.

### 4.10. Western-Blot Assay

The cells (5 × 10^6^ cells per dish) were seeded on 100-mm cell culture dishes with 10 mL medium, incubated for 24 h, treated with the medium containing the samples at dose level of 25 mg/mL for 24 h, harvested by trypsin-EDTA, washed three times with the cold PBS, and lysed on ice for 30 min with 100 μL the RIPA Lysis Buffer supplemented with 1 mmol/L PMSF. The lysate was centrifuged at 12,000× *g* at 4 °C for 5 min. The supernatant was collected as total cellular protein. Then, protein content was measured using the BCA Protein Assay Kit. Protein (20 μg) of total protein extracts were separated on a 10−15% SDS-PAGE gel and transferred to the PVDF membrane. The blots were blocked with 5% BSA, probed with the primary anti-body (dilution 1:3000) in blocking buffer at 4 °C overnight. The bands were incubated with the anti-rabbit secondary anti-body horseradish peroxidase conjugate. The enhanced chemiluminescence was covered on the PVDF membrane, and the signal was detected using a Chemi Scope 6300 (Clinx Science Instrument, Shanghai, China).

### 4.11. Statistical Analysis

All data from three independent experiments were analyzed by the SPSS 16.0 software (SPSS Inc., Chicago, IL, USA) and one-way analysis of variance (ANOVA) with Duncan’s multiple range tests, and expressed as means or means ± standard deviations.

## 5. Conclusions

This study found that Cu^2+^ and especially Mn^2+^ fortification of a peptic bovine lactoferrin hydrolysate BLH led to desired changes for its in vitro anti-cancer effects on human gastric cancer BGC-823 cells. Compared with BLH itself, the Cu/Mn fortified BLH had increased growth inhibition, arrested more cells in the G0/G1-phase, disrupted mitochondrial membrane greatly, and promoted cell apoptosis. Furthermore, Cu/Mn fortification led to expression changes of seven apoptosis-related proteins in the cells, and thereby triggered cell apoptosis via the mitochondrial pathway. Mn^2+^ was always more efficient than Cu^2+^ to increase these assayed activities, while higher metal level consistently resulted in enhanced activities. Fortification of trace metal ions thus suggests endowing BLH with increased anti-cancer action in the BGC-823 cells.

## Figures and Tables

**Table 1:** Chemical features of the bovine LF and prepared BLH (dry basis).

Samples	Protein (g/kg)	−NH_2_ (mmol/g Protein)	Degree of Hydrolysis (%)	Fe (mg/kg)
LF	957.3 ± 3.1	0.49 ± 0.01	0	140.6 ± 2.8
BLH	923.4 ± 2.7	0.93 ± 0.03	5.1 ± 0.1	130.3 ± 3.3

## Abbreviations

BLH	Bovine lactoferrin hydrolysate
CCK-8	Cell counting kit-8
DMEM	Dulbecco’s modified Eagle’s medium
EDTA	Ethylenediamine tetra-acetic acid
FBS	Fetal bovine serum
5-FU	5-Fluorouracil
LF	Lactoferrin
MMP	Mitochondrial membrane potential
PBS	Sodium phosphate buffered solution
PI	Propidium iodide
PMSF	Phenylmethanesulfonyl fluoride

## References

[1] Bhat Z.F., Kumar S., Bhat H.F. (2015). Bioactive peptides of animal origin: a review. J. Food Sci. Tech..

[2] Chalamaiah M., Kumar B.D., Hemalatha R., Jyothirmayi T. (2012). Fish protein hydrolysates: proximate composition, amino acid composition, antioxidant activities and applications: a review. Food Chem..

[3] García M.C., Puchalska P., Esteve C., Marine M.L. (2013). Vegetable foods: a cheap source of proteins and peptides with antihypertensive, antioxidant, and other less occurrence bioactivities. Talanta.

[4] Reddy G.V., Friend B.A., Shahani K.M., Farmer R.E. (1983). Antitumor activity of yogurt components. J. Food Protect..

[5] Sheih I.C., Fang T.J., Wu T.K., Lin P.H. (2010). Anticancer and antioxidant activities of the peptides fraction from algae protein waste. J. Agric. Food Chem..

[6] Xu X.X., Jiang H.R., Li H.B., Zhang T.N., Zhou Q., Liu N. (2010). Apoptosis of stomach cancer cell SGC-7901 and regulation of Akt signaling way induced by bovine lactoferrin. J. Dairy Sci..

[7] Pan W.R., Chen P.W., Chen Y.L., Hsu H.C., Lin C.C., Chen W.J. (2013). Bovine lactoferricin B induces apoptosis of human gastric cancer cell line AGS by inhibition of autophagy at a late stage. J. Dairy Sci..

[8] Chea C., Miyauchi M., Inubushi T., Ayuningtyas N.F., Subarnbhesaj A., Nguyen P.T., Shrestha M., Haing S., Ohta K., Takata T. (2018). Molecular mechanism of inhibitory effects of bovine lactoferrin on the growth of oral squamous cell carcinoma. PLoS ONE.

[9] Wakabayashi H., Bellamy W., Takase M., Tomita M. (1992). Inactivation of Listeria monocytogenes by lactoferricin, a potent antimicrobial peptide derived from cow’s milk. J. Food Protect..

[10] Shin K., Yamauchi K., Teraguchi S., Hayasawa H., Tomita M., Otsuka Y., Yamazak S. (1998). Antibacterial activity of bovine lactoferrin and its peptides against *Enterohaemorrhagic Escherichia coli* O157: H7. Lett Appl. Microbiol..

[11] Zhao H.J., Zhao X.H. (2018). Modulatory effect of the supplemented copper ion on in vitro activity of bovine lactoferrin to murine splenocytes and RAW264.7 macrophages. Biol. Trace Res..

[12] Li S.T., Zhou H.B., Huang G.R., Liu N. (2009). Inhibition of HBV infection by bovine lactoferrin and iron-, zinc-saturated lactoferrin. Med. Microbiol. Immun..

[13] Bogden J.D. (2000). The Essential Trace Elements and Minerals.

[14] Mertz W. (1981). The essential trace elements. Science.

[15] Nordberg M., Nordberg G.F. (2016). Trace element research-historical and future aspects. J. Trace Elem. Med. Biol..

[16] Beard J.L. (2001). Iron biology in immune function, muscle metabolism and neuronal functioning. J. Nutr..

[17] Tisato F., Marzano C., Porchia M., Pellei M., Santini C. (2010). Copper in diseases and treatments, and copper-based anticancer strategies. Med. Res. Rev..

[18] Leng J., Shang Z.M., Quan Y. (2018). High energy transition state complex increased anticancer activity: a case study on Cu^II^-complexes. Inorg. Chem. Commun..

[19] Zhou D.F., Chen Q.Y., Qi Y., Fu H.J., Li Z., Zhao K.D., Gao J. (2011). Anticancer activity, attenuation on the absorption of calcium in mitochondria, and catalase activity for manganese complexes of N-substituted di(picolyl)amine. Inorg. Chem..

[20] Bo L.Y., Li T.J., Zhao X.H. (2018). Copper or magnesium supplementation endows the peptic hydrolysate from bovine lactoferrin with enhanced activity to human gastric cancer AGS cells. Bio. Trace Elem. Res..

[21] Duarte D.C., Nicolau A., Teixeira J.A., Rodrigues L.R. (2011). The effect of bovine milk lactoferrin on human breast cancer cell lines. J. Dairy Sci..

[22] Chi C.F., Hu F.Y., Wang B., Li T., Ding G.F. (2015). Antioxidant and anticancer peptides from the protein hydrolysate of blood clam (Tegillarca granosa) muscle. J. Funct. Foods.

[23] Pan X., Zhao Y.Q., Hu F.Y., Chi C.F., Wang B. (2016). Anticancer activity of hexapeptide from skate (Raja porosa) cartilage protein hydrolysate in Hela cells. Mar. Drugs.

[24] Arias M., Hilchie A.L., Haney E.F., Bolscher J.G., Hyndman M.E., Hancock R.E., Vogel H.J. (2017). Anticancer activities of bovine and human lactoferricin-derived peptides. Biochem. Cell Biol..

[25] Chalamaiah M., Yu W., Wu J. (2018). Immunomodulatory and anticancer protein hydrolysates (peptides) from food proteins: A review. Food Chem..

[26] Guedes J.P., Pereira C.S., Rodrigues L.R., Cortereal M. (2018). Bovine milk lactoferrin selectively kills highly metastatic prostate cancer PC-3 and osteosarcoma MG-63 cells *in vitro*. Front Oncol..

[27] Tomita M., Wakabayashi H., Shin K., Yamauchi K., Yaeshima T., Iwatsuki K. (2009). Twenty-five years of research on bovine lactoferrin applications. Biochimie.

[28] Freiburghaus C., Janicke B., Lindmark-Mansson H., Oredsson S.M., Paulsson M.A. (2009). Lactoferricin treatment decreases the rate of cell proliferation of a human colon cancer cell line. J. Dairy Sci..

[29] Farhan M., Khan H.Y., Oves M., Al-Harrasi A., Rehmani N., Arif H., Hadi S.M., Ahmad A. (2016). Cancer therapy by catechins involves redox cycling of copper ions and generation of reactive oxygen species. Toxins.

[30] Farhan M., Oves M., Chibber S., Hadi S.M., Ahmad A. (2017). Mobilization of nuclear copper by green tea polyphenol epicatechin-3-gallate and subsequent prooxidant breakage of cellular DNA: implications for cancer chemotherapy. Int. J. Mol. Sci..

[31] Amin A.R., Kucuk O., Khuri F.R., Shin D.M. (2009). Perspectives for cancer prevention with natural compounds. J. Clin. Oncol..

[32] Mao X.Y., Gu J.N., Sun Y., Xu S.P., Zhang X.Y., Yang H.Y., Ren F.Z. (2009). Anti-proliferative and anti-tumour effect of active components in donkey milk on A549 human lung cancer cells. Int. Dairy J..

[33] Yang J.I., Tang J.Y., Liu Y.S., Wang H.R., Lee S.Y., Yen C.Y., Chang H.W. (2016). Roe protein hydrolysates of giant grouper (Epinephelus Lanceolatus) inhibit cell proliferation of oral cancer cells involving apoptosis and oxidative stress. Biomed. Res. Int..

[34] Ouyang L., Shi Z., Zhao S., Wang F.T., Zhou T.T., Liu B., Bao J.K. (2012). Programmed cell death pathways in cancer: a review of apoptosis, autophagy and programmed necrosis. Cell Proliferat..

[35] Hung C.C., Yang Y.H., Kuo P.F., Hsu K.C. (2014). Protein hydrolysates from tuna cooking juice inhibit cell growth and induce apoptosis of human breast cancer cell line MCF-7. J. Funct. Foods.

[36] Kluck R.M., Bossy-Wetzel E., Green D.R., Newmeyer D.D. (1997). The release of cytochrome c from mitochondria: a primary site for Bcl-2 regulation of apoptosis. Science.

[37] Hollstein M., Sidransky D., Vogelstein B., Harris C.C. (1991). P53 mutations in human cancers. Science.

[38] Donovan M., Cotter T.G. (2004). Control of mitochondrial integrity by Bcl-2 family members and caspase-independent cell death. Bioch. Bioph. Acta.

[39] Yang T., Zhu H., Zhou H., Lin Q.L., Li W.J., Liu J.W. (2012). Rice protein hydrolysate attenuates hydrogen peroxide induced apoptosis of myocardiocytes H9c2 through the Bcl-2/Bax pathway. Food Res. Int..

[40] Wang L., Zhang J., Yuan Q., Xie H., Shi J., Ju X. (2016). Separation and purification of an anti-tumor peptide from rapeseed (Brassica campestris L.) and the effect on cell apoptosis. Food Funct..

[41] Dai F., Wang Q., Fan G.J., Du Y.T., Zhou B. (2018). Ros-deriven and preferential killing of HepG2 over L-02 cells by a short-term cooperation of Cu(Ⅱ) and a catechol-type reveratrol analog. Food Chem..

[42] Qi X.M., He L.L., Zhong H.Y., Distelhors C.W. (1997). Baculovirus p35 and z-VAD-fmk inhibit thapsigargin-induced apoptosis of breast cancer cells. Oncogene.

[43] Bellamy W., Takase M., Wakabayashi H., Kawase K., Tomita M. (1992). Antibacterial spectrum of lactoferricin B, a potent bactericidal peptide derived from the N-terminal region of bovine lactoferrin. J. Appl. Bacterial.

[44] AOAC (2005). Official Methods of Analysis of Association of Official Analytical Chemists International.

[45] Church F.C., Swaisgood H.E., Porter D.H., Catignani G.L. (1983). Spectrophotometric assay using *o*-phthaldialdehyde for determination of proteolysis in milk and isolated milk proteins. J. Dairy Sci..

[46] Nagy A., Marciniak-Darmochwal K., Krawczuk S., Gelencser E. (2009). Influence of glycation and pepsin hydrolysis on immunoreactivity of albumin/globulin fraction of herbicide resistant wheat line. Czech J. Food Sci..

